# Development and validation of a sleep questionnaire, SNoRE 3.0, to evaluate sleep in companion dogs

**DOI:** 10.1038/s41598-023-40048-1

**Published:** 2023-08-16

**Authors:** A. Mondino, C. Ludwig, C. Menchaca, K. Russell, K. E. Simon, E. Griffith, A. Kis, B. D. X. Lascelles, M. E. Gruen, N. J. Olby

**Affiliations:** 1 Department of Clinical Sciences, College of Veterinary Medicine, North Carolina State University, Raleigh, NC 27606 USA; 2 Department of Statistics, North Carolina State University, Raleigh, NC 27606 USA; 3 Institute of Cognitive Neuroscience and Psychology, Research Centre for Natural Sciences, Budapest, Hungary; 4 Translational Research in Pain, Department of Clinical Sciences, College of Veterinary Medicine, North Carolina State University, Raleigh, NC 27606 USA

**Keywords:** Medical research, Neurology

## Abstract

Disturbances in the sleep–wake cycle are a debilitating, yet rather common condition not only in humans, but also in family dogs. While there is an emerging need for easy-to-use tools to document sleep alterations (in order to ultimately treat and/or prevent them), the veterinary tools which yield objective data (e.g. polysomnography, activity monitors) are both labor intensive and expensive. In this study, we developed a modified version of a previously used sleep questionnaire (SNoRE) and determined criterion validity in companion dogs against polysomnography and physical activity monitors (PAMs). Since a negative correlation between sleep time and cognitive performance in senior dogs has been demonstrated, we evaluated the correlation between the SNoRE scores and the Canine Dementia Scale (CADES, which includes a factor concerning sleep). There was a significant correlation between SNoRE 3.0 questionnaire scores and polysomnography data (latency to NREM sleep, ρ = 0.507, *p* < 0.001) as well as PAMs’ data (activity between 1:00 and 3:00 AM, *p* < 0.05). There was a moderate positive correlation between the SNoRE 3.0 scores and the CADES scores (ρ = 0.625, *p* < 0.001). Additionally, the questionnaire structure was validated by a confirmatory factor analysis, and it also showed an adequate test–retest reliability. In conclusion the present paper describes a valid and reliable questionnaire tool, that can be used as a cost-effective way to monitor dog sleep in clinical settings.

## Introduction

Sleep is a fundamental physiological process that emerged early in animal evolution^1,2^. It has several critical functions such as replenishment of energy stores, physical restoration and growth, elimination of accumulated waste products, and facilitation of memory consolidation and learning^3–5^. Sleep deprivation weakens the immune system response, increases perception of pain^6,7^, induces metabolic alterations^8^ and impairs physical and neurocognitive performance^9–11^. Despite sleep’s evident role in both human and non-human well-being, simple sleep measurement tools are scarce, especially in veterinary settings.

Dogs spend an average of 10 h per day sleeping and as diurnal animals, most of their sleep occurs between 9 PM and 6 AM with occasional naps/inactivity in the afternoon^12–14^. They can suffer from sleep disorders such as narcolepsy, REM sleep behavioral disorder and sleep apnea and chronic painful diseases such as osteoarthritis can cause sleep disruption^15,16^. Changes in sleep also occur with aging in dogs, with older dogs showing sleep fragmentation at night and increased sleep episodes during the day^17^. Moreover, senior dogs are prone to developing canine cognitive dysfunction syndrome (CCDS), a neurodegenerative disease that shares several similarities with Alzheimer’s disease^18,19^. This syndrome is characterized by a constellation of neurobehavioral alterations including changes in sleep–wakefulness cycle^18,20^.

The gold standard technique to study sleep is polysomnography (PSG), a simultaneous recording of the electroencephalogram (EEG), electromyogram (EMG), and electrooculogram (EOG)^21–23^. In dogs, a non-invasive PSG technique has been developed and validated making it possible to determine how much of the recorded time dogs spend awake, in drowsiness, and in Non-REM (NREM) and REM sleep^24^. However, the use of PSG in the clinical setting can be quite challenging, since it is time consuming, requires expensive equipment and specific expertise. In addition to this, a handler needs to remain with the dog during the procedure to make sure that they do not remove the electrodes.

Another approach to measure the rest/activity patterns in dogs is the use of collar-mounted physical activity monitors (accelerometers, PAMs)^14,15^. Physical activity monitors are considered a valid tool in sleep medicine and research as they have been proven to accurately detect sleep-periods when compared to PSG^25,26^. However, while PAMs are becoming more accessible with respect to cost, they are still used mainly in research settings. Additionally, analysis of the data obtained from the PAMs is time consuming and while some PAMs offer automatic analyses through specific software, this has not yet been validated in dogs^27,28^. There is a need for an easy to administer, low-cost, validated tool to evaluate sleep in dogs to complement more objective sleep assessments. Sleep questionnaires [also known as clinical metrology instruments (CMIs)] are used commonly in human medicine to provide a subjective measure of sleep quality^29,30^. Studies in humans have shown sleep questionnaire scores are correlated with sleep time and sleep efficiency measured by both PSG and PAMs^31,32^. In veterinary medicine, CMIs completed by owners and caregivers are widely used in clinical settings and research to measure a variety of variables^33–38^. A sleep questionnaire, the sleep and nighttime restlessness evaluation (SNoRE) has been used in pain research in dogs but has not yet been validated against PSG, and while it captured a reduction in nighttime restlessness in dogs treated for osteoarthritis pain, its findings did not correlate with activity monitor data^15,39^. The aim of this work was to modify and validate SNoRE by comparing its performance to PSG recordings and PAMs.

## Materials and methods

All study protocols were conducted with the approval of the NCSU Institutional Animal Care and Use Committee (protocol numbers 21-303, 21-396 and 21-376) and all methods were carried out in accordance with relevant guidelines and regulations. Owners of the dogs who participated in this study reviewed and signed an informed consent form. Methodology is reported in accordance with the ARRIVE Guidelines. The NCSU Institutional Review Board determined that humans were not used in this research because all data collected pertained to dogs, not humans. This work was categorized as “Not Human Subject Research”.

### Population of dogs

We used three different populations of dogs, senior dogs participating in a longitudinal study of neuroaging, senior dogs participating in a clinical trial (data collected from baseline, before any treatment) and a group of healthy adult dogs. Dogs were considered senior if they were older than 75% of their expected lifespan and adult if they were older than 1 year and younger than 75% of their expected lifespan. Expected lifespan was calculated with a formula that uses the dogs’ height and weight^40^. We ask the owners to bring the dogs for a first screening visit. To be included in the study, senior dogs had to be systemically healthy, food motivated and amenable to handling for the EEG evaluation. Dogs exhibiting aggressive behavior or showing higher levels of anxiety during the screening visit were not included. Other exclusion criteria included recent initiation (defined as less than 4 weeks) of psychoactive medications that might alter behavioral testing, and severe mobility or visual impairment that prevented cognitive testing. Beyond age, there were no other criteria for inclusion or exclusion of adult dogs. We did not exclude any dog based on their breed, sex, or spay/neuter status. Dogs were recruited by advertising through the College of Veterinary Medicine, NCSU and social media.

### Questionnaire design

The questionnaire was designed by modifying the existing SNoRE questionnaire. The original questionnaire consists of 6 items that ask the owners to rate the frequency with which their dog moves, twitches, dreams, shifts position, vocalizes, and paces at sleep time over the last 7 days on a scale from 1 to 10. Since twitching and vocalizations tend to occur during REM sleep in dogs, there is overlap between this question and the question about the frequency of dreaming. Moreover, our aim was to generate a questionnaire that captured disruptions to sleep. Therefore, we changed the questions about twitching and vocalizations to “*how much did vocalizations affect your dog's sleep over the last 7 days”* and *“how much did twitching affect your dog's sleep over the last 7 days”*. Sleep questionnaires in human medicine usually include questions about breathing difficulties and snoring during sleep to capture sleep apnea associated problems^40,41^. As dogs can also suffer from sleep apnea^42,43^, we included questions about the frequency of snoring and the frequency of breathing interruptions. One of the main reasons for dogs to wake up at night is to eliminate, therefore we included a question about the frequency of elimination during the night. Finally, as difficulty sleeping could induce daytime sleepiness, we included a question about the frequency of naps during the day. The modified questionnaire thus consisted of 9 items (Supplementary file 1).

An exploratory factor analysis was performed to determine how many components explained the variance of the modified SNoRE (mSNoRE). This analysis was performed using maximum likelihood as the factoring method and varimax rotation. Factors were retained if they were composed of at least two items and if they met the Kaiser’s criteria rule (eigenvalue > 1.0)^44,45^. Once the factors were selected, internal consistency was evaluated in each factor by calculating its Cronbach’s alpha coefficient. Values of 0.70 or higher were considered satisfactory^46,47^.

### Criterion validity: polysomnography recordings

**Figure 1:**
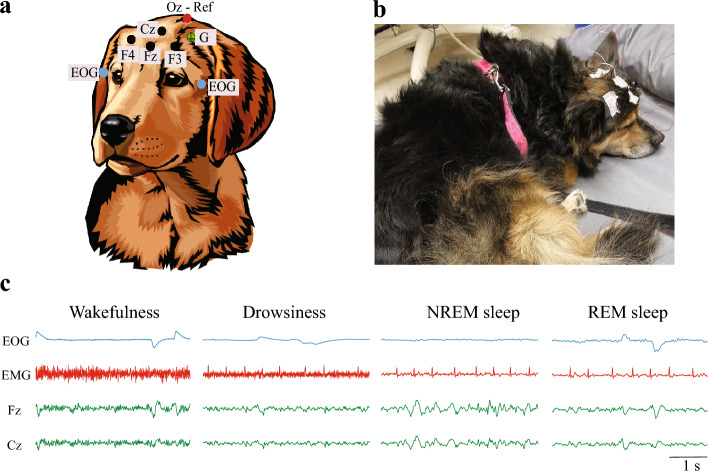
Polysomnography recordings. (**a**) Electrode location used to record the electroencephalogram (EEG) and electrooculogram (EOG). Active electrodes (black circles) were placed at left, right and central frontal cortices (F3, F4, Fz) and at central parietal (Cz), and a reference electrode (red circle) was placed at the occipital protuberance (Oz). Electrodes used for EOG (light blue circles) were placed at the left and right zygomatic arch near the lateral canthus of each eye. Electromyography (EMG) was measured using two electrodes placed over the neck muscles. A ground electrode (green circle) was located over the left parietal area. (**b**) Picture of a dog sleeping during a PSG recording. (**c**) Representative recordings showing each behavioral state. Wakefulness was recognized by high frequency activity in the EEG, high tone in the EMG and numerous high amplitude eye movements. Drowsiness was defined by fast EEG activity, reduced but observable tone in the EMG and decreased amplitude and frequency of eye movements. NREM sleep was recognized by high amplitude low frequency EEG, mainly delta (1–4 Hz). The EMG in this state was decreased and no or occasional low amplitude eye movements. Finally, REM sleep was characterized by high frequency activity in the EEG, muscle atony in the EMG and rapid eye movements that were also seen as artifacts in the EEG.

We determined the criterion validity of the mSNORE by comparing the scores to the data obtained by means of two standard techniques used to evaluate the sleep–wakefulness cycle, PSG and PAMs. For PSG recordings we included senior dogs participating in the longitudinal study of neuroaging as well as young adult dogs. We first performed a 30 min adaptation recording to acclimate the dog to the recording room and setup. After 1–7 days, the dogs returned, and we performed a 2-h long PSG recording during an afternoon nap. Recordings were always started between 12:30 and 1:30 PM. Owners brought their dogs’ bed or blankets to both the adaptation and the actual recording, to increase dog comfort. Recordings were performed in a quiet room, with dim light and computer-generated white noise. Room temperature was maintained at 20 °C. We followed a previously described protocol^48^ with the addition of 2 active electrodes and changing the location of the EMG electrodes from the lumbar area to the neck (Fig. 1a,b). We used reusable gold-coated electrodes (Genuine Grass, Natus Medical Incorporated, WI, USA) which were held in place with SAC2 electrode cream (Cadwell Laboratories, WA, USA) after preparing the skin with a skin preparation and electrode solution (Signaspray, Parker Laboratories, NJ, USA). Recordings were performed with Cadwell Easy II software (Cadwell Laboratories, WA, USA). Electrode impedance was maintained under 20 kΩ. The recordings were obtained using a frequency sample of 200 Hz, and high and low pass filters of 0.53 Hz and 70 Hz. A notch filter was also used to remove 60 Hz power-line noise. States of wakefulness, drowsiness, NREM and REM sleep were manually scored in 5 s epochs using Spike 2 software, version 9.04b (Cambridge Electronic Design, Cambridge, UK) with criteria described previously^24^ and illustrated in Fig. 1c. Latency to drowsiness, NREM and REM sleep as well as percentage of total time recorded spent in wakefulness, drowsiness, NREM and REM sleep were calculated. Additionally, sleep efficiency was calculated as the sum of the time spent in NREM and REM sleep divided by the total time recorded.

### Criterion validity: physical activity monitors

Senior dogs participating in both the longitudinal study of neuroaging and the clinical trial were included. A PAM (Actical monitor; Phillips Respironics, OR, USA) was mounted to the dogs’ collar between 1 and 2 weeks after owners completed the questionnaire. This is a uniaxial accelerometer that has been shown to be a valid surrogate measure of activity in dogs^49^. The sampling rate was set to 30 Hz, and epoch length was 60 s. Owners were instructed to keep the collar with the PAM on their dog over a 2-week period. Data were downloaded using Actical Software version 3.11 (Phillips Respironics, OR, USA). Daily activity graphs were inspected, and days that had complete lack of activity (counts of 0) for more than 3 h during the day were excluded from the analysis. Data from the first acceptable 5 weekdays were used in the analysis. We only evaluated weekdays since it has been demonstrated that dog’s activity differs between weekdays and weekends^14,50^.

### Correlation with canine cognitive dysfunction

We have recently demonstrated in senior dogs a negative correlation between their sleep time and their cognitive function^51^. Therefore, senior dogs with cognitive impairment were expected to have higher mSNoRE values. We asked owners of senior dogs to complete the Canine Dementia Scale (CADES), a validated CMI used to evaluate the presence of behaviors associated with CCDS^37^. This questionnaire is divided into four factors, one of them is the “sleep–wakefulness cycle subscale” which asks two specific questions about sleep.

### Confirmatory factor analysis and test–retest reliability

In the second phase of the study, we confirmed the factor structure in a larger, independent sample using confirmatory factor analysis. We calculated the following goodness of fit measures: chi-square (χ^2^); root mean square error of approximation (RMSEA) and the comparative fit index (CFI). The chi-square is also known as the “lack of fit index” because it is used to reject the null hypothesis of a perfect fit, therefore, the lower the chi-square value relative to the degrees of freedom and the higher the *p* value, the better model fit^52^. The RMSEA evaluates the overall model against the observed data and values of 0.05 or lower are considered an indicator of good model fit^53,54^. Finally, the CFI assesses the discrepancy and determines the relative improvement between the data and the proposed model. Values greater than 0.95 were considered indicators of good model fit^53,54^.

For test–retest reliability, 2 weeks after the first measurement, we re-sent the questionnaire to the same group of owners who completed the questionnaire for the confirmatory factor analysis. We evaluated the test–retest reliability using an intraclass correlation coefficient (ICC), with a two-way random effects, single rater type and consistency definition i.e., ICC (3,1)^55^.

### Statistical analysis

Variables analyzed were tested for normality by means of Shapiro–Wilk test. Data that were not normally distributed were analyzed with non-parametric tests.

To determine criterion validity, we determined the association between the mSNORE and PSG measurements as well as the PAMs’ data. We determined Spearman rank-order correlations between mSNoRE scores and latency to drowsiness, to NREM and REM sleep, sleep efficiency, and percentage of time in wakefulness, drowsiness, NREM and REM sleep. In these analyses *p* values ≤ 0.05 were considered statistically significant. In addition to this, we determined the association between sleep questionnaires and activity using the package “Actigraphy” (version 1.4.0) in Rstudio version 2023.03.0 (Posit Software PBC MA, USA)^14,56,57^. Using the raw per-minute activity counts we calculated values for the average per-minute activity for each minute over a 24-h period. Thus, each per-minute average reflected the mean activity over the 5 days analyzed for that particular timepoint in a day. The Actigraphy package uses functional linear modeling (FLM) to create a smoothed curve of activity through a Fourier expansion model. It also performs a non-parametric permutation F-test that allows us to compare activity levels in dogs with different scores in the sleep questionnaire. For this analysis we used 1000 permutations. We used point-wise critical values (a curve with the F permutation proportion at each time point) and a global critical value (single number referring to the proportion of maximized F values from each permutation)^57^ to determine significance. Since the global critical value is more robust, we used this as a threshold for significant differences in activity^56,57^. Additionally, we evaluated the correlation between the mSNoRE, and total CADES score as well as the sleep–wakefulness subscale. Since CADES scores are correlated with age^58,59^, and aging itself could also be affecting the sleep–wakefulness cycle^17^, we used partial Spearman rank-order correlations controlling by age.

## Results

### Population

For the initial questionnaire structure development and validation, 68 dog owners completed the sleep questionnaire. Median age of the dogs was 12.5 years (range 1.3–16.2), 55.8% (n = 38) were castrated males and 44.2% (n = 30) were spayed females. Twenty-seven dogs (39.7%) were mix-breed dogs and there were 8 American pitbull terriers, 7 Labrador retrievers, 4 golden retrievers, 3 Australian shepherds and 3 basset hounds, 2 beagles, 2 border collies, 2 Pomeranians, and 1 each of Brittany spaniel, collie, dachshund, German shorthaired pointer, Maltese, Rhodesian ridgeback, Irish setter and Shih Tzu. Thirty-eight of these dogs participated in the polysomnography study, 41 wore PAMs and 57 owners of these dogs completed the CADES questionnaire. All the dogs that completed the CADES questionnaire were senior dogs.

**Figure 2:**
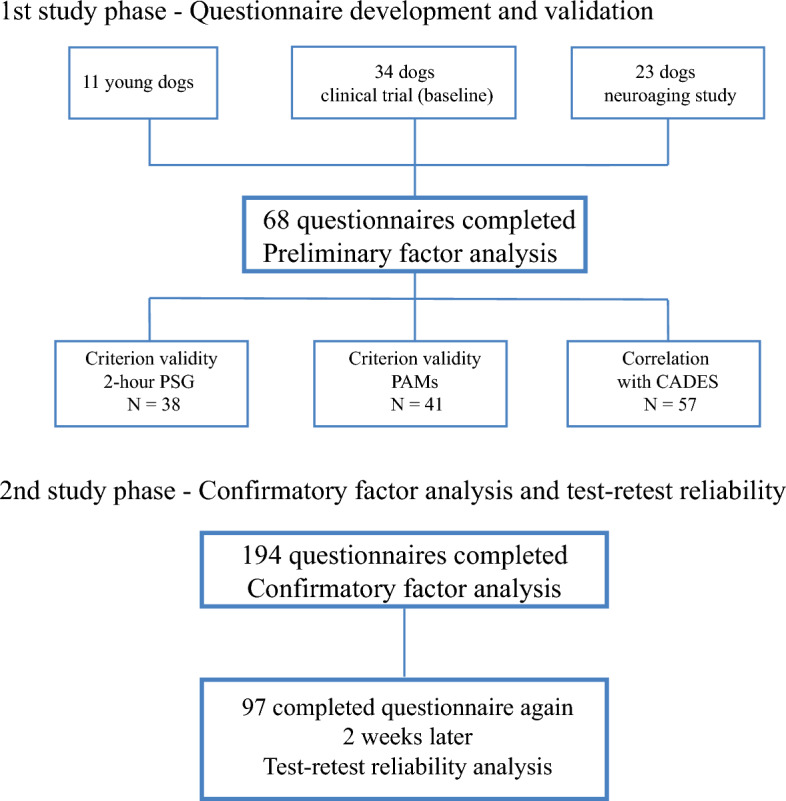
Study design and population. Schematic representation of the population of dogs used in each part of the study. *CADES* Canine Dementia Scale, *PSG* polysomnography, *PAMs* physical activity monitors.

For the second phase of the study (confirmatory factor analysis), 194 owners completed the questionnaire, the median age of the dogs was 6.90 years (range 0.43–17.02). However, 9 owners did not report their dog’s age and we did not collect information about the dogs’ breed. Ninety-seven of these owners completed the questionnaire again 2 weeks later, data that were used to evaluate the test–retest reliability. This population of dogs had a median age of 7.2 years old (range 0.69–16.96), with one owner not reporting the age of their dog. Figure 2 schematizes the population of dogs used in each part of the study. All data analyzed in this study are provided in Supplementary data files 2–4.

### Internal structure

The question about frequency of dreaming was not included in the final analysis of the sleep questionnaire since the questionnaire’s goal is to detect sleep disruptions and dreaming is normal and expected during sleep. However, we looked at results of this question separately to evaluate if there was a correlation between the owners’ perception of their dog’s dreaming time and the time in REM sleep measured by PSG.

Principal component analysis found 3 components with eigenvalues over 1. However, 1 of them was composed of only 1 question, and a 2-factor solution was deemed most appropriate. An exploratory factor analysis identified that 2 factors were sufficient to explain the full dimensionality of the data (χ^2^ = 19.85, *p* = 0.10). These 2 factors accounted for 42.9% of the variance. The first factor included questions about sleep quality; Q1 (Ability to sleep at bedtime), Q2 (Ability to sleep continuously during the night), Q3 (Frequency of eliminations during the night), and Q8 (Frequency of breathing interruptions during sleep). This factor explained 23.4% of the variance. The second factor included questions about how dreaming may interrupt sleep; Q5 (how frequently vocalizations wake the dog up) and Q6 (how frequently twitching wakes the dog up). This factor accounted for 19.5% of the variance. Two additional questions [Q7 (frequency of snoring) and Q9 (frequency of naps during the day)] did not have enough factor loading to be included in either of the factors. Cronbach’s alpha showed an acceptable internal consistency of the items within factor 1 (0.73). Deletion of any item had a minimal effect of Cronbach’s alpha (maximum 0.02). Items included in factor 2 also had good internal consistency with a Cronbach’s alpha of 0.86.

**Figure 3:**
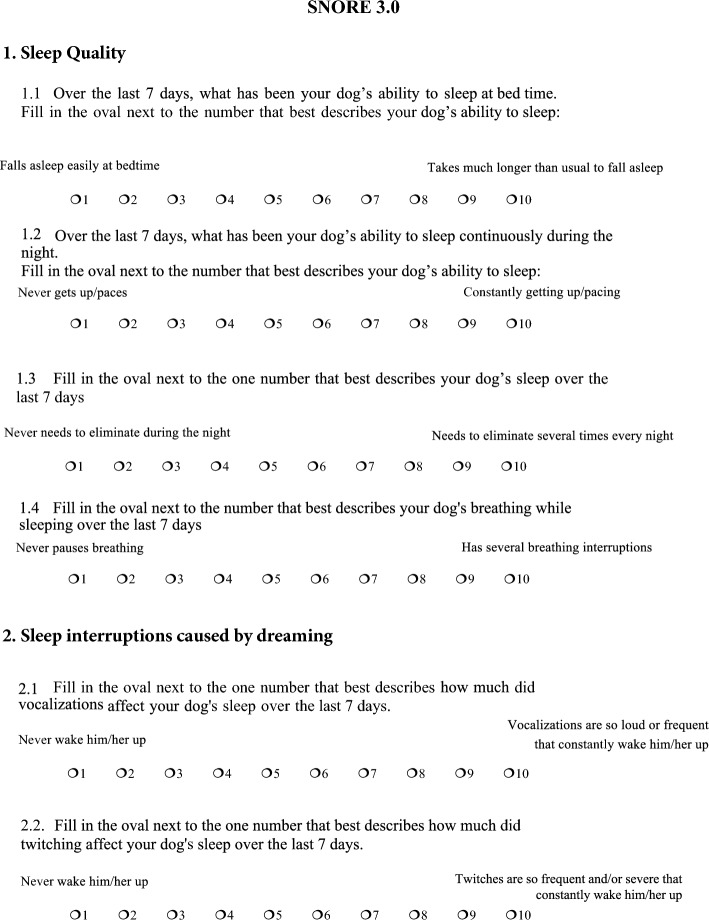
SNORE 3.0, final version of the questionnaire. The questionnaire is composed of two different factors; “sleep quality” and “sleep interruptions caused by dreaming”.

Therefore, the final SNoRE questionnaire (named SNoRE 3.0) retained 6 questions assigned to Factor 1 which was named “Sleep Quality” and Factor 2 named “Sleep interruptions caused by dreaming” (Fig. 3). For the 68 dogs included in the first phase of the study, the median total value of SNoRE 3.0 was 9, (range 6–30), with a median of 6 (range 4–26) for Factor 1 and a median of 2 (range 2–12) for Factor 2.

### Criterion validity

#### Correlation with polysomnography recordings

Two-hour PSG recordings were performed in 38 dogs. Median SNoRE 3.0 score in this subset of dogs was 7.5 (range 6–25). On PSG, 36 dogs entered drowsiness, 34 NREM sleep and 24 REM sleep. Dogs spent a median of 52.3% (range 16.5–100) of the total recording time in wakefulness, 21.3% (0–63.3) in drowsiness, 19.3% (0–52.9) in NREM sleep and 2.1% (0–28.9%) in REM sleep. Median sleep efficiency was 22.4% (0–69.7%). Median latency to drowsiness was 6.1 min (1.0–74.4), latency to NREM sleep was 23.05 min (3.3–81.2) and latency to REM sleep was 34.2 min (5.8–96.7).

**Figure 4:**
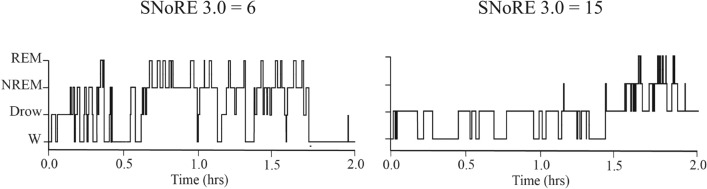
Representative hypnograms of a dog with the lowest possible SNoRE 3.0 score and with a relatively high SNoRE score. Drow: drowsiness; W: wakefulness

Figure 4 shows 2 representative hypnograms from dogs with a low (6) and relatively high (15) SNoRE 3.0 score. Results of the correlation analyses between the SNoRE 3.0 questionnaire scores and the sleep architecture variables are shown in Table 1. We found a significant moderate^60^ positive correlation between latency to NREM sleep and the SNoRE 3.0 score. There was also a weak negative correlation between % of time in NREM sleep and SNoRE 3.0 score that trended toward significance.

**Table 1:** Correlation analysis between total SNoRE 3.0 scores and Sleep architecture variables measured by polysomnographic recordings. Statistically significant correlations are indicated by an *.

	Total		Factor 1		Factor 2	
	Spearman ρ	*p* value	Spearman ρ	*p* value	Spearman ρ	*p* value
Latency to drowsiness (min)	0.103	0.548	0.241	0.156	− 0.104	0.544
Latency to NREM sleep (min)	0.507	0.003*	0.545	0.001*	0.210	0.234
Latency to REM sleep (min)	0.139	0.516	0.154	0.471	− 0.130	0.545
Time in wakefulness (%)	0.167	0.315	0.253	0.125	0.024	0.887
Time in drowsiness (%)	− 0.120	0.471	− 0.185	0.267	− 0.047	0.778
Time in NREM sleep (%)	− 0.301	0.066	− 0.308	0.060	− 0.136	0.414
Time in REM sleep (%)	− 0.204	0.219	− 0.239	0.147	− 0.085	0.614
Sleep efficiency (%)	− 0.268	0.103	− 0.276	0.093	− 0.143	0.393

Finally, we evaluated the correlation between Q4 (frequency of dreaming) and percentage of time in REM sleep and REM sleep latency, but no correlation was found (ρ = − 0.019, *p* = 0.909 and ρ = − 0.039, *p* = 0.857, respectively).

#### Association with activity levels over time measured by PAMs

**Figure 5:**
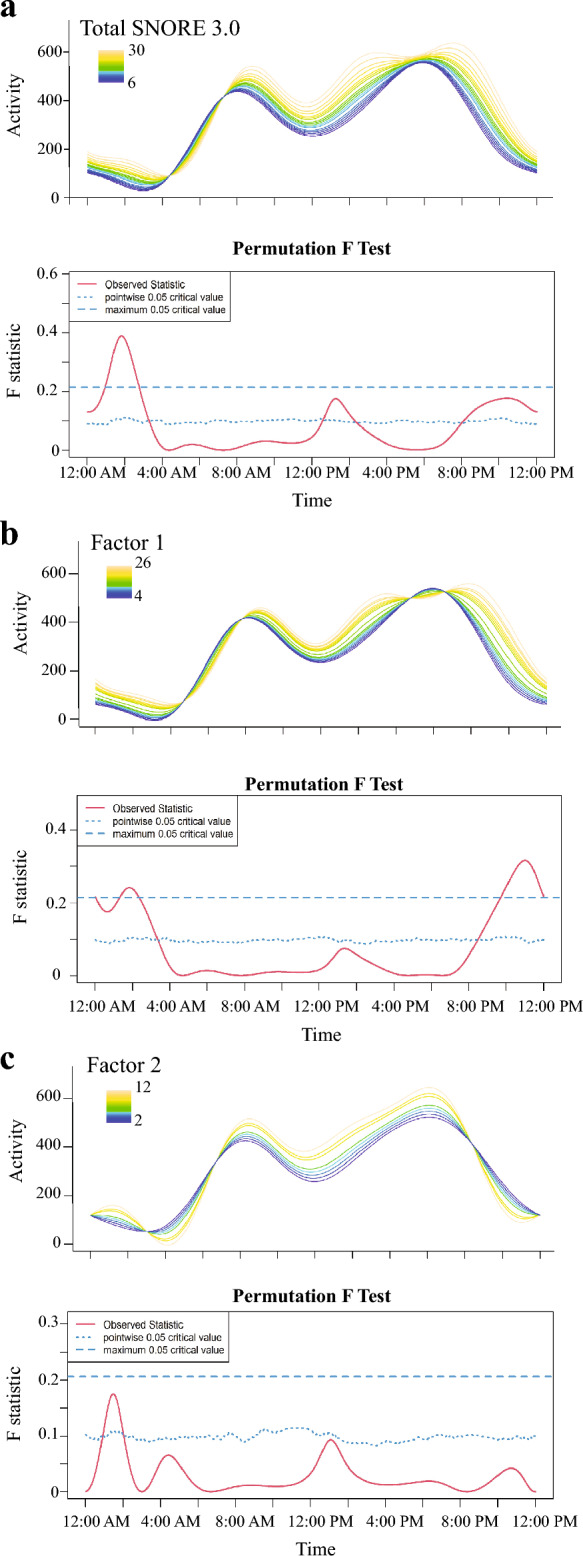
Relationship between the total SNoRE 3.0 (**a**), factor 1 (**b**) and factor 2 (**c**) and activity levels as measured by the PAM. The upper graph shows activity counts over time for different levels of the sleep questionnaire scores (color coded) and the lower graph indicates the level of observed statistic (red line). When the red line is above the test of significance set at 0.05 (blue lines) it indicates significant differences between different levels of the variable. The dashed and the dotted lines indicate global and point-wise tests of significance respectively.

Forty-one dogs wore a PAM for at least 1 week. Median total SNoRE 3.0 score for this subset of dogs was 10 (range 6–30). Median score for factor 1 was 7 (range 4–26) and for factor 2 was 2 (range 2–12). Results of the FLM analysis are shown in Fig. 5. We found a significant association between the total SNoRE 3.0 score and activity between 1:00 and 3:00 AM, which showed that dogs with higher scores were more active during this time (Fig. 5a). When looking at each factor individually, dogs with higher scores on factor 1 were also more active during that period of time, but the results only achieved statistical significance around 2:00 AM. Moreover, dogs with higher scores were significantly more active between 9:00 PM and 12:00 AM (Fig. 5b). On the other hand, while dogs with higher scores on factor 2 tended to be more active between 1:00 and 2:00 AM, these did not reach the threshold for global statistical significance (Fig. 5c).

### Correlation with CADES

**Figure 6:**
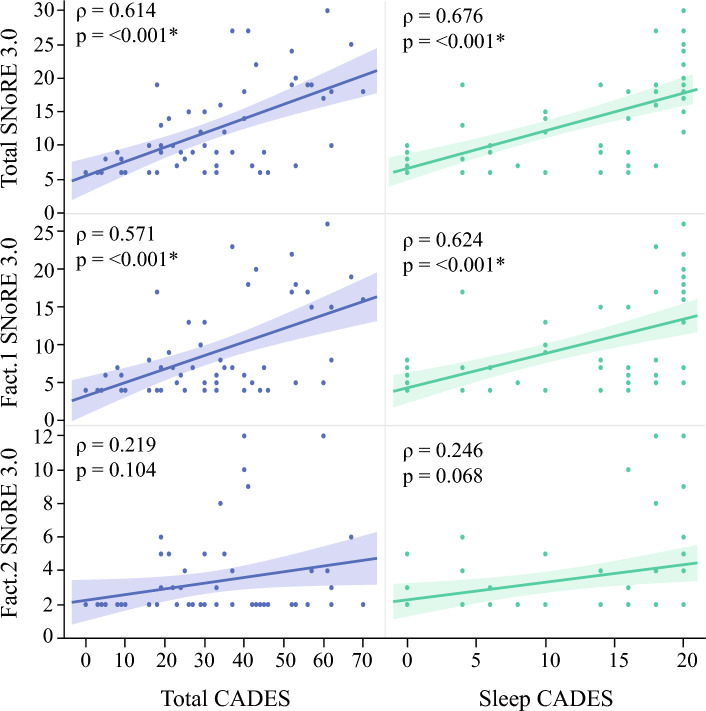
Correlations between SNoRE 3.0 (total score and the two individual factors) and CADES (total score and the sleep–wakefulness subscale). The rho and *p* values represents the results of the partial correlation analysis controlling for the effect of age.

The CADES questionnaire was completed by 57 of the 68 owners who completed the mSNoRE questionnaire in the first phase of the study. Median CADES score was 30 (range 0–70), and the Sleep subscale of the CADES score was 10 (range 0–20). The median total SNoRE 3.0 for these dogs was 9 (range 6–30), with a median of 6 (range 4–26) for Factor 1 and 2 (range 2–12) for factor 2. As shown in Fig. 6, after correcting by age, total SNoRE 3.0 and factor 1 showed moderate positively correlations with both the total CADES score and the sleep-subscale of the CADES (*p* < 0.05). Factor 2 was not correlated with either total CADES score or the sleep-subscale. Correlations were stronger with the CADES sleep-subscale than with the total CADES score; among these, total SNoRE 3.0 showed the highest correlation coefficient.

### Confirmatory factor analysis

**Figure 7:**
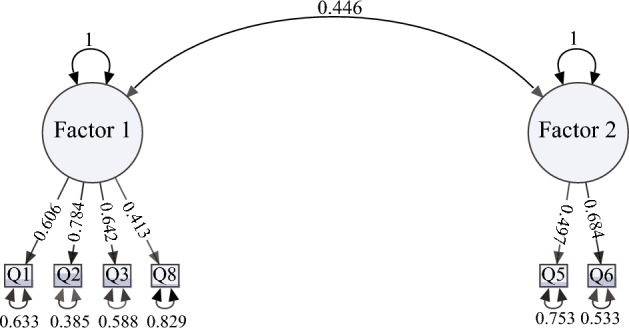
Path diagram output of the confirmatory factor analysis of the two factor-solution of the SNoRE 3.0 with standardized parameters estimates.

The path diagram showing the factor structure is shown in Fig. 7. The two-factor structure model showed an excellent fit: Χ^2^_(8)_ = 7.36, *p* = 0.498; RMSEA = 0.0000 [0.0000–0.0798]; CFI = 1.000.

### Test–retest reliability

The total SNoRE 3.0 showed good test–retest reliability (ICC _(3,1)_ = 0.817 (0.738–0.874) *p* ≤ 0.001). Individually, factor 1 also showed good reliability (ICC _(3,1)_ = 0.817 (0.738–0.874) *p* ≤ 0.001) while factor 2 showed moderate reliability (ICC _(3,1)_ = 0.674 (0.549–0.770) *p* ≤ 0.001).

## Discussion

In this study we established and validated an owner-based sleep questionnaire that can provide a low-cost, easy to apply and reliable measure of sleep quality in dogs. This questionnaire was developed by modifying the existing SNoRE questionnaire^39^. While the SNoRE questionnaire detected analgesia-associated improvement in night-time sleep quality in osteoarthritic dogs, it was not yet validated for internal consistency, test–retest reliability, or in a population other than dogs with osteoarthritis. Also, this first version of the SNoRE questionnaire did not seem to be in full agreement with objective (PAMs) data^39,61^. The original SNoRE questionnaire had three questions related to dreaming, “How much did your dog twitch, how much did your dog dream, and how much did your dog vocalize while sleeping?”. It has been shown that during REM sleep (sleep stage in which most dreams occur) dogs can exhibit rhythmic paddling of limbs and can whine, yelp and produce muffled barking^62,63^. However, while dogs can experience spontaneous arousals after REM sleep, dreaming constitutes a natural part of sleep and mild twitching, or vocalizations may occur without disrupting sleep. Therefore, in this new sleep questionnaire we asked the owners how often twitching or vocalizations actually interrupt their dog’s sleep and removed the question about frequency of dreaming from the analysis. This becomes particularly important if we consider that, while uncommon, dogs can suffer from REM sleep behavioral disorder, which is characterized by violent limb or face movements and vocalizations that can fragment sleep^16,63,64^. Moreover, during the questionnaire design process, we added questions regarding breathing-related sleep characteristics that were not included in the SNoRE questionnaire, i.e., frequency of snoring and breathing pauses. These questions were included because dogs, particularly brachycephalic breeds, can suffer from sleep breathing disorders that can also lead to hypoxemia and consequent arousals^42,43,65,66^. The final version (SNoRE 3.0) retained the question about breathing interruptions but the question about frequency of snoring was removed by the factor analysis. The question about snoring was introduced because it can be associated with sleep apnea in both humans and dogs. However, snoring can occur with or without associated sleep apnea^67,68^. Finally, since night-time sleep disruptions can lead to increased sleepiness throughout the day, we included a question about the number of naps during the day. This question was also discarded by the factor analysis and was not included in the final version of the questionnaire. We hypothesize that it is challenging for owners to provide accurate responses to this question given many dogs are not observed during the day. The final SNoRE 3.0 questionnaire was composed of 6 questions divided into two factors, and this structure was validated by a confirmatory factor analysis. This questionnaire showed an adequate test–retest reliability.

We demonstrated that the questionnaire has a good construct validity by showing a high correlation between its scores and the scores in the total score of the CADES questionnaire, as well as in the sleep–wakefulness subscale. CADES is used in veterinary medicine to detect behavioral signs compatible with CCDS which is characterized by changes in the sleep–wakefulness cycle among other behavioral alterations such as disorientation, changes in house soiling behavior and social interaction, anxiety, learning and memory impairment^37^. This questionnaire has been validated using biomarkers of neurodegeneration^69,70^ and with performance in cognitive tests^20,71^. Although the sleep–wakefulness aspect is taken into consideration in this questionnaire, the specific subscale consists of only two questions, “How often does your dog have abnormal behavior at night (waking up, wandering, vocalizing)” and “How often does your dog have difficulty falling asleep or sleeping excessively”^37^. Therefore, we believe that the sleep–wakefulness subscale of the CADES questionnaire does not consider other potential sleep changes such as night-time elimination needs, parasomnias or breathing abnormalities, which has been addressed by our questionnaire.

Additionally, we demonstrated that the total SNoRE 3.0, as well as the factor 1 have good criterion validity by comparing them to the most accurate tools to measure sleep, PSG and PAMs. We found that dogs with higher scores have more difficulty falling asleep (evidenced by a longer latency to NREM sleep). However, we did not find a correlation between the time spent in each behavioral state and the sleep questionnaire scores. This could be the result of many factors. First, we performed the PSG recordings during the afternoon, when dogs usually take naps, and we only recorded 2 h. This was done because overnight recordings require significantly more effort from the owners and the researchers (a handler needs to stay with the dog checking that the dog does not pull or remove the electrodes), and shorter afternoon recording sessions are much more achievable in veterinary settings unless a wireless polysomnographic equipment is available^72^. In addition to this, the PSG recordings were performed at the College of Veterinary Medicine, and owners answered the questionnaire based on the dog’s behavior at home. In this regard, Bunford et al.^73^ have demonstrated that there is an effect of both the time of day as well as the sleep location on the sleep architecture. Functional linear model analysis of the PAMs data allowed us to look at differences in activity between dogs with different sleep scores at different times of the day. PAMs are becoming increasingly popular in veterinary research^14,15,74^ and while NREM and REM sleep cannot be differentiated by these devices, and quiet wakefulness can be miscoded as sleep^75^, studies in humans and in narcoleptic dogs have shown PAMs to have a reasonable accuracy in detecting sleep–wakefulness periods when comparing them to PSG recordings^25,26,76^. Higher total SNoRE 3.0 scores were significantly associated with higher activity between 1:00 and 3:00 AM, demonstrating the ability of the owners to detect and report restless sleep at night. Similar results were found with factor 1 alone, but the period of time in which the F-statistic surpassed the global level of significance was shorter (around 1:45–2:15 AM). There was a tendency for dogs with higher scores to be more active between 1:00 and 2:00 AM, but non statistically significant differences were observed when evaluating factor 2 alone. These results suggest that the combination of both factors (total score) is a  better predictor of sleep-time activity, than each factor individually. However, factor 1 alone was better than the total score in detecting differences in dogs from 9:00 PM to 12:00 AM. We were not able to demonstrate the construct and criterion validity of factor 2 alone; this specific factor is focused on sleep interruptions caused by dreaming, and therefore, the performance of this factor alone should be tested on dogs that suffer from parasomnias such as REM sleep disorders. Importantly, including factor 2 did not prevent the total scores from showing good validity.

We acknowledge some limitations of this study that warrant consideration. While we have tested the test–retest reliability, we have not specifically assessed the interobserver reliability. This aspect is important to investigate since the questionnaire is completed by pet owners who may not have any veterinary or medical knowledge. However, the likelihood is that different owners will have very different perspectives on their dog’s sleep because of differing relationships with the dogs and different personal sleep habits. As such, it is vital that the same person always completes the questionnaire in longitudinal studies. Future studies should determine whether two different owners of the same dog would provide similar responses when completing the questionnaire, particularly considering owners with different education or background. Another limitation of the study is that although we had some young adult dogs in the sample, the studied population was mainly composed of senior dogs. Therefore, future studies should evaluate the criterion validity of this sleep questionnaire in a higher number of dogs from all life stages. However, we consider that having a questionnaire that is validated in older dogs is extremely useful since they are prone to develop sleep–wakefulness cycle disturbances^19,37,77,78^. Similarly, there were very few brachycephalic dogs in this study, subsequent research should evaluate the performance of the SNoRE 3.0 in this specific population due to their high prevalence of sleep apneas^65^. Additionally, to avoid the first-night effect^48^, we performed only a single adaptation recording, and dogs may benefit from additional adaptation sessions prior to the actual recording session. Future studies should evaluate whether additional adaptation sessions improve the correlation between the SNoRE 3.0 and PSG. Moreover, we did not test this questionnaire in dogs diagnosed with sleep disorders such as sleep apnea or REM sleep behavioral disorders, and we consider that should be a next step in its validation. In conclusion, we developed a modified version of the SNoRE questionnaire that can be used as an easy to apply, low-cost and reliable tool to evaluate sleep disturbances in dogs.

## Conclusions

The SNoRE 3.0 questionnaire can be used as an easy to apply, low-cost and reliable tool to evaluate sleep disturbances in dogs. Future studies should look at the performance of this questionnaire in a larger number of dogs, including all life stages and dogs with diagnosed sleep disorders.

### Supplementary Information

Supplementary Information 1.

Supplementary Information 2.

Supplementary Information 3.

Supplementary Information 4.

## Data Availability

All data analyzed in this study are provided in Supplementary data files 2–4.
