# Supplementary material for: Development and validation of a sleep questionnaire, SNoRE 3.0, to evaluate sleep in companion dogs
Source: Sci Rep. 2023 Aug 16;13:13340. doi: 10.1038/s41598-023-40048-1 (PMC10432410; doi:10.1038/s41598-023-40048-1)
Supplement: Supplementary file 1 — Supplementary Information 1. [file 41598_2023_40048_MOESM1_ESM.pdf]

Supplementary File 1: The original version of the SNoRE questionnaire used in this study

Sleep Questionnaire

1. Over the last 7 days, what has been your dog's ability to sleep at bed time.
Fill in the oval next to the number that best describes your dog's ability to sleep:

Falls asleep easily at bedtime — Takes much longer than usual to fall asleep

❍1 ❍2 ❍3 ❍4 ❍5 ❍6 ❍7 ❍8 ❍9 ❍10

2. Over the last 7 days, what has been your dog's ability to sleep continuously during the night.
Fill in the oval next to the number that best describes your dog's ability to sleep:

Never gets up/paces — Constantly getting up/pacing

❍1 ❍2 ❍3 ❍4 ❍5 ❍6 ❍7 ❍8 ❍9 ❍10

3. Fill in the oval next to the one number that best describes your dog's sleep over the last 7 days

Never needs to eliminate during the night — Needs to eliminate several times every night

❍1 ❍2 ❍3 ❍4 ❍5 ❍6 ❍7 ❍8 ❍9 ❍10

4. Fill in the oval next to the one number that best describes your dog's sleep over the last 7 days

Never dreams — Constantly dreaming (vocalizing/twitching)

❍1 ❍2 ❍3 ❍4 ❍5 ❍6 ❍7 ❍8 ❍9 ❍10

5. Fill in the oval next to the one number that best describes how much vocalizations affected your dog's sleep over the last 7 days.

Never wake him/her up — Vocalizations are so loud or frequent that they constantly wake him/her up

❍1 ❍2 ❍3 ❍4 ❍5 ❍6 ❍7 ❍8 ❍9 ❍10

6. Fill in the oval next to the one number that best describes how much twitching affected your dog's sleep over the last 7 days.

Never wake him/her up — Twitches are so frequent and/or severe that constantly wake him/her up

❍1 ❍2 ❍3 ❍4 ❍5 ❍6 ❍7 ❍8 ❍9 ❍10

Sleep Questionnaire

7. Over the last 7 days how much has your dog snored at night?

Never snores | Constantly snoring

❍1 ❍2 ❍3 ❍4 ❍5 ❍6 ❍7 ❍8 ❍9 ❍10

8. Fill in the oval next to the number that best describes your dog's breathing while sleeping over the last 7 days

Never pauses breathing | Has several breathing interruptions

❍1 ❍2 ❍3 ❍4 ❍5 ❍6 ❍7 ❍8 ❍9 ❍10

9. Fill in the oval next to the one number that best describes your dog’s sleep during the day time over the last 7 days in comparison with 6 months ago

Has slept similar number of naps | Has increased considerably the number and duration of naps

❍1 ❍2 ❍3 ❍4 ❍5 ❍6 ❍7 ❍8 ❍9 ❍10
